# Anticipatory Behaviour During the Approach to Feeding Times as a Measure of Horse Welfare

**DOI:** 10.3390/ani14243677

**Published:** 2024-12-20

**Authors:** Fernando Mata, Georgina Boyton, Tamsin Young

**Affiliations:** 1 Centre for Research and Development in Agrifood Systems and Sustainability, Instituto Politécnico de Viana do Castelo, Rua da Escola Industrial e Comercial Nun’Alvares 34, 4900-347 Viana do Castelo, Portugal; 2 School of Life Science, Wrexham Glyndŵr University Northope Campus, Holywell Road, Northop, Mold CH7 6AA, UK

**Keywords:** ad libitum feeding, animal welfare, arousal behaviour, circadian rhythms, equine, judgement bias, rationed feeding, stereotypic behaviour

## Abstract

Anticipatory behaviour is increasingly being recognised as a measure of animal welfare. This behaviour is linked to reward sensitivity, which reflects the balance of positive and negative experiences. This study examined anticipatory behaviour in horses fed either ad libitum or rationed diets, aiming to identify differences in behaviour patterns during the periods of one hour immediately before and after feeding. Behavioural data were collected via video surveillance over five days, focusing on the pre- and post-feeding periods of stabled horses. The data were successfully fit to Poisson models. The results indicate that ration-fed horses exhibit more stereotypic and arousal behaviours, with anticipatory behaviour intensifying closer to feeding times. These findings suggest a potential link between feeding stress and anticipatory behaviour, especially in horses on rationed diets. This study underscores the importance of considering feeding practices in horse welfare management and highlights anticipatory behaviour as a valuable indicator for assessing animal well-being. Such assessments, rooted in circadian rhythms, offer a less resource-intensive means for ongoing welfare monitoring in animal care settings.

## Simple Summary

Anticipatory behaviour, which reflects animals’ excitement or stress about upcoming rewards, is gaining attention in the assessment of animal welfare. This study looked at how horses’ behaviour changes around feeding time, comparing horses on free-access (ad libitum) diets with those on limited (rationed) diets. Using video monitoring, researchers observed horses one hour before and after feeding over five days. The results showed that horses on restricted diets displayed more signs of stress and repetitive actions, especially as feeding time approached. These behaviours suggest that limiting food might cause feeding-related stress, which shows up in the horses’ anticipation behaviours. The findings point to the need for thoughtful feeding practices in horse care to improve welfare, suggesting that monitoring these behaviours is an effective, low-cost way to keep track of animals’ well-being over time.

## 1. Introduction

Judgement bias [1,2] and anticipatory behaviour [3,4] have been suggested as animal welfare-graded measures, indicating the spectrum of an animal’s subjective assessment of their well-being ranging from favourable to unfavourable conditions. These behaviours are believed to be connected to reward sensitivity, which reflects the overall balance of positive and negative experiences for individual animals [3]. Judgment bias assessments examine an animal’s perception of ambiguous stimuli, with an animal approaching such stimuli labelled as “optimistic” and one avoiding them as “pessimistic” [5]; anticipatory behaviours occur before an anticipated favourable event and are expected to be more pronounced in individuals with fewer opportunities for desirable experiences [4].

Anticipatory behaviour in animals has been referred to as a conditioned response formed from cues given by the trainer [6] or, in the instance of this study, the caregiver, and it is associated with reward sensitivity [7]. Within many species, including equines, the learning theory is defined as a process of adaptive changes to behaviour because of previous experiences [8]. When a positive reward is incorporated into a training or husbandry routine, it is expected to elicit anticipatory behaviour [9].

Wild horses, like the majority of herbivorous animals, are natural prey. As such, the equine digestive system has evolved to require a relatively small stomach due to the high frequency of small meals [6], and alterations in these habits in domestic horses create a risk of gastrointestinal disorders [10]. Stabled horses’ diets are routinely maintained by the caregiver, and horses may lack sufficient foraging opportunities, suffering negative effects on the digestive system [11]. Though it may be unnatural to a horse’s environmental and physiological needs to be fed a rationed diet, there are reasons for horses to be fed this way: horses suffering from conditions that require a calorie-controlled diet to reduce and regulate glucose levels will require a rationed feeding regime [12]. However, stabled horses are often fed at the convenience of the caregiver due to personal routines and commitments, resulting in gastrointestinal stress [13] and the development of stereotypies [14].

Studies have shown that horses anticipate feeding times due to their natural circadian rhythm [15,16,17,18]. There is an argument that anticipatory behaviour can have positive and negative effects on the physiological and psychological state, depending on the environment that this behaviour is presented within [9,19]. Often, in the domesticated horse environment, natural needs are not met. The differences between the domestic and the feral horse environments are substantial. It is questioned that an increase in anticipatory behaviour seen during feeding times is due to the horses’ need to achieve natural environmental needs [20]. Peters and colleagues [19] have presented evidence that horse anticipation increases in the time between human cues and feed delivery. The question is yet to be answered if there is a significant timescale to the onset of anticipatory behaviour before regular feeding times.

Within this cross-sectional study [21], anticipatory behaviour in a group of horses was monitored to compare behaviours in horses rationed or ad libitum-fed to evaluate the onset of anticipatory behaviour in the approach to regular feeding time.

It is hypothesised that significantly different anticipatory behaviours would develop within different periods of time, in pre-feeding and post-feeding periods. A second hypothesis is raised for significant differences in anticipatory behaviour in horses fed a rationed forage diet in comparison to horses fed an ad libitum forage diet.

## 2. Materials and Methods

### 2.1. Data Collection and Ethogram Production

We used (n = 6) horses in this study. Subjects were all stabled in the same yard for a minimum of 14 months and were stabled (3.66 × 3.66 m stables) individually within one outside yard structure with visibility of each other. All stalls were joined as part of one yard in the order of subject numbers. At the time of this study, all subjects were fed haylage of either ad libitum or a rationed amount and had access to water ad libitum.

Each horse’s feeding schedule consisted of a morning feed prepared and delivered by the research staff. The subjects, transitioning from 24 h summer turnout in October 2022, were provided with one to three hours of daily turnout and received concentrate feed twice a day by the research staff.

The feed provided by the research staff consisted of 1 kg of Dengie^®^ Healthy Hoof Chaff (molasses-free), delivered at 06:30 h and again at 16:30 h. This feeding schedule remained consistent throughout the observation period. In the evenings, horses were also fed by their owners following their own routines and using individually selected feed types.

During the observation period, all horses were given haylage at 16:30 h by the researcher. Horses were brought in daily in the order of subjects 5 through 1 and were fed using hay nets prepared by their owners. While the quantity of hay in the nets was not recorded, all horses received the same hay net each night. For those on ad libitum feeding, the same hay net was provided regardless of any leftover hay in their stall. Conversely, ration-fed horses typically had no hay remaining when their evening net was delivered.

Hay nets for post-turnout feeding were prepared in advance by the owners or livery yard staff. Throughout both the observation period and the 28 days preceding it, all feeding by the researcher adhered to this schedule.

No human presence was made in the yard until three minutes before the arrival of feed to minimise anticipation presented from human cues.

Horses were stabled most of the time, with a daily 3 h turnout period each. They were always stabled between 12:00 a.m. and 7:00 a.m., plus additional hours, except during their turnout. The turnout timing varied daily and was not specified due to differences in owners’ and the yard’s routines. During the observations, horses were turned out for 3 h per day between 07:00 and 12:00. Turnout was managed either by livery yard staff or individual owners, and the specific timing varied within this period. All winter paddocks, approximately four acres each, were shared by four horses, though not always simultaneously due to individual turnout schedules. The paddocks had grass available for grazing but no hay feeders. Automatic water troughs were installed in all paddocks. The winter paddocks were fully fenced and had no trees.

Data collected were used to produce ethograms, structured as per Table 1. Behaviours are grouped into categories, and in each category, the specific behaviour elements are described. The behaviour elements considered are independent of each other, and to the set of behavioural elements, the option “others” was also added to meet the exhaustion rule.

The term “husbandry” refers to equine management practices, which vary depending on the purpose for which the horses are maintained, such as work, sport, or leisure, and the specific conditions under which they are kept. “Buck” describes a springing motion where the horse raises its hindquarters off the ground, sometimes accompanied by a longitudinal twisting of the body. “Pawing” refers to the act of striking a vertical or horizontal surface, or the air, with a forelimb. “Kicking” involves one or both hind legs being thrust backwards or sideways, often making contact with part of the stable or other nearby objects. “Striking” refers to a forelimb being lifted upward and forward, usually directed at an object, another horse, or a person, and then forcibly placed back onto the ground. “Stamping” involves a single fore or hind limb being lifted slightly off the ground and quickly placed back down with force. The distinction between stamping and pawing lies in their movement patterns and purposes. Stamping is typically a single or rapid successive motion of the fore or hind limbs, often associated with a mild threat or protest, such as dislodging flies from the legs. In contrast, pawing is carried out only by the forelegs, involves slower, repeated movements and may serve different functions. In domestic horses, pawing has been identified as either a displacement behaviour when the horse is restrained or as an operantly conditioned response when the horse anticipates food. “Foraging for food” in the stable refers to the search for food and feeding on the forage provided in the hay net. The hay net is an enrichment to promote foraging behaviour. To distinguish between a relaxed and a tense horse, the following behaviours were considered. Relaxed horse: ears moving casually back and forth, eyes soft and blinking, licking and/or chewing. Tense horse: ears held stiff, tight muscles, fixed gaze and showing whites of the eyes.

During the observation periods (one hour pre feeding and one hour post feeding), the number of behaviours performed was registered.

The observation periods were divided into three distinct phases: baseline, pre feeding and post feeding. The pre-feeding period refers to one hour immediately before feeding, while the post-feeding period relates to one hour following feeding. The baseline period covers the time between feeding sessions, excluding the pre-feeding and post-feeding periods.

Behaviours were recorded via video surveillance, using Reolink^®^ Go 4G LTE (Reolink, Los Angeles, CA, USA) mobile security cameras. This method was selected to exclude possible human interaction influencing behaviours during observation. Cameras were positioned in each stable giving a full view of the box, high enough to ensure no interference from the horse. Cameras were installed into boxes two days before data collection to allow horses to habituate. Behaviours were recorded for five days (a total of 12 h per subject) from one hour pre feeding until one hour post feeding using an ethogram structure. Two baseline observations were also recorded from each subject during a time when anticipation was predicted to be at a low level (one hour post turnout, supplied with forage).

### 2.2. Data Analysis

The ethogram produced counts of behaviours performed during the observation period. Feeding period (pre, post or baseline) and feeding types (ad libitum- or ration-fed) were used as independent variables. Behaviour counts were modelled to differentiate between feeding periods and feeding types in each one of the behaviour groups, using Generalised Linear Models from the Poisson family. We tested log-linear and negative binomial links and chose the best fit using Akaike’s information criterion (AIC).

In a Poisson regression model, the predicted counts of events in the dependent variable can be computed following the parameterisation of the model as

(1) $$\lambda_{i}=e^{\beta_{1}x_{1}+\beta_{2}x_{2}+\beta_{3}x_{3}+\ldots +\beta_{n}x_{n}}$$

where *β_i_* are the parameters of the fitted model, and *x_i_* are the dummy variables associated with the levels of the factors of the independent variable considered.

A further analysis was produced to study the evolution of behaviours in the pre-feeding period. The one-hour observation period was divided into three 20 min sub-periods, and these sub-periods were then contrasted with repeated measures ANOVA aiming to identify significant differences signalling an eventual evolutive pattern with the approach of the feeding time. The post hoc test used was the Least Significant Difference (LSD). The prerequisites for the ANOVA were checked via Levene’s test for the homogeneity of variances and through Q-Q plots to evaluate the distribution of the residuals.

The software used in all the tests was the statistical package IBM Corp.^®^ SPSS^®^ Statistics, version: 29.0.0.0 (241), Armonk, NY, USA.

## 3. Results

The Poisson regressions were successfully fit to the different behaviour groups, and the most parsimonious models were achieved with a log-linear link. Table 2 summarises the statistics and degree of adjustment of the models, and Table 3 gives the parameterisation of the models. Table 4 shows the means for the different levels of factors considered in this study (ad libitum- and ration-fed for feed type and baseline, pre feeding and post feeding for feeding period) together with their respective interactions.

As can be observed in Table 4, for the stereotypical behaviour model, the results show that stereotypies are much more frequent in ration-fed horses when compared with ad libitum-fed horses. There are no significant differences in stereotypic behaviour in the pre- and post-feeding periods; however, both these periods show a higher frequency of these displays when compared to the baseline. Considering the interactions, a higher frequency of displays is observed in the post-feeding period of ration-fed horses.

Concerning the arousal behaviour model, this type of behaviour has higher frequencies in the post-feeding period of ration-fed horses.

The investigation behaviour model has higher frequencies both in the pre-feeding period for ad libitum-fed horses and in the pre- and post-feeding periods of ration-fed horses.

The locomotion is more frequent in the pre- and post-feeding periods of ad libitum-fed horses and in the pre-feeding period of ration-fed horses.

Finally, concerning the maintenance group of behaviours, the baseline period shows significantly higher frequencies when compared to the pre- and post-feeding periods.

The results obtained after the analysis of the evolution of the different behaviour groups through time in the hour before feeding, displayed in Table 5, are as follows:

There is no significant difference through time in the stereotypical behaviour in relation to ad libitum-fed horses, while in ration-fed horses, we can observe that the frequency increases as the time of feeding approaches;

Concerning the arousal behaviours, there is a positive evolution of the behaviours as the feeding time approaches for both the ad libitum- and the ration-fed horses, however, more intense in ration-fed horses;

For the stationary behaviour, we observe that the frequency increases as the feeding time approaches for both the ad libitum- and the ration-fed horses, however, more intense in ration-fed horses;

There are no significant differences concerning the investigation behaviour;

As opposed to the stationary behaviour, locomotion decreases as the feeding time approaches for both groups of horses but more intensively in ration-fed horses;

Finally, concerning the maintenance behaviours, their frequency decreases equally in both groups of horses as the feeding time approaches.

**Table 4:** Mean values of the number of observations in the different group behaviours calculated after the models in Table 2. Different letters in superscript are indicative of significant differences (*p* < 0.05).

Group Behaviour	Independent Variables and Interaction					
	Feed Type		Feed Period		F. Type × F. Period	
Stereotypical	AL	1.78 ^a^	Base	2.25 ^a^	AL × Base	0.33 ^a^
	Rationed	16.28 ^b^	Pre	8.29 ^b^	AL × Pre	6.87 ^b^
			Post	8.34 ^b^	AL × Post	2.47 ^c^
					Rationed × Base	15.25 ^d^
					Rationed × Pre	10.00 ^b^
					Rationed × Post	28.20 ^e^
Arousal	AL	0 ^a^	Base	0 ^a^	AL × Base	0 ^a^
	Rationed	1.60 ^a^	Pre	0.82 ^a^	AL × Pre	0.3 ^a^
			Post	1.26 ^b^	AL × Post	1 ^b^
					Rationed × Base	0.50 ^ab^
					Rationed × Pre	1.60 ^b^
					Rationed × Post	5.10 ^c^
Investigation	AL	13.02 ^a^	Base	5.29 ^a^	AL × Base	3.50 ^a^
	Rationed	18.97 ^b^	Pre	30.53 ^c^	AL × Pre	33.53 ^d^
			Post	24.02 ^b^	AL × Post	18.80 ^c^
					Rationed × Base	8.00 ^b^
					Rationed × Pre	27.80 ^d^
					Rationed × Post	30.70 ^d^
Locomotion	AL	1.07 ^a^	Base	0 ^a^	AL × Base	0.17 ^b^
	Rationed	0 ^a^	Pre	3.58 ^c^	AL × Pre	3.67 ^c^
			Post	1 ^b^	AL × Post	2.00 ^c^
					Rationed × Base	0 ^a^
					Rationed × Pre	3.50 ^c^
					Rationed × Post	0.50 ^b^
Maintenance	AL	9.74 ^a^	Base	57.70 ^c^	AL × Base	56.67 ^e^
	Rationed	31.85 ^a^	Pre	16.00 ^a^	AL × Pre	13.13 ^a^
			Post	31.57 ^b^	AL × Post	3 ^d^
					Rationed × Base	75 ^e^
					Rationed × Pre	0 ^b^
					Rationed × Post	28.20 ^c^

Note: in feeding type, “ad libitum-fed” was used as the response and “ration-fed” as the reference; in feeding period, “post” and “baseline” were used as the response and “pre” as the reference; AL, ad libitum-fed.

**Table 5:** Evolution of behaviours one hour pre feeding. The one-hour observation period was divided into three 20 min sub-periods: 41–60, 21–40 and 0–20 min before feeding. Observed mean number of behaviour displays for ad libitum-fed and ration-fed horses.

	Feeding Type	Ad Libitum				Ration-Fed			
Group Behaviour		*p*-Value	41–60	21–40	0–20	*p*-Value	41–60	21–40	0–20
Stereotypies		<0.05	0.8	0.4	0.2	<0.01	2.2 ^a^	3.8 ^a^	7.2 ^b^
Arousal		<0.05	14.4 ^a^	17.8 ^a,b^	22.4 ^b^	<0.01	25 ^a^	27.4 ^a^	33.6 ^b^
Stationary		<0.01	4 ^a^	8.8 ^b^	12.75 ^c^	<0.001	7 ^a^	11.8 ^b^	18.4 ^c^
Investigation		>0.05	2.2	1.4	2	>0.05	4.8	4.4	5.8
Locomotion		<0.05	3.2 ^a^	4 ^a^	0.6 ^b^	<0.001	18.4 ^a^	4.2 ^b^	1.2 ^c^
Maintenance		<0.05	4 ^a^	4 ^a^	1 ^b^	<0.05	4 ^a^	3.6 ^a^	1 ^b^

Notes: The *p*-values refer to the repeated measures ANOVA test; different letters in superscript are indicative of significant differences (*p* < 0.05).

## 4. Discussion

This study aimed to compare anticipatory behaviour across all time measurements to identify the onset of anticipatory behaviour during the approach to regular feeding and to identify if there was a difference in anticipatory behaviour between horses fed an ad libitum forage diet and horses fed a rationed forage diet.

Horses are herbivorous, gregarious animals and evolved using two-thirds or more of their time budget grazing and walking in groups in pursuit of herbage [18,22,23]. As such, it is expected that domestic horses without access to a paddock with grazeland may find an alternative to satisfy their innate behaviour. Several authors have discussed the causes of stereotypies in horses, and the lack of opportunity to express the feeding instinct has been pointed out as one of the reasons [16,18,24].

There is previous evidence that horses fed within a rigid routine for years are more likely to exhibit stereotypic behaviour as the feeding time approaches [22,25]. Nevertheless, a study indicates that no behavioural differences are felt if the horses are completely restricted to their box and not allowed to graze or interact with peers regularly [17], which is not the case in our study. These authors suggest that horses severely restricted in their movements, diet and social contact with peers may have the deterioration of their welfare state limited if bedded in straw, allowed to interact with peers through grilled windows between paddocks and the concentrate feeding periods are increased. As such, it is hypothesised that horses that are turned out regularly (as those in our study) may benefit from husbandry practices like those described in the study of Ruet and colleagues [17].

To our knowledge, no reports exist suggesting that the stereotypic behaviours may extend even further in the post-feeding period of concentrate ration-fed horses. One of the main theories explaining the development of stereotypies during the feeding period is the gastrointestinal irritation caused by the low pH formation in the stomach that is not balanced by the production of saliva observed in grazing horses. While grazing and ingesting roughage, horses produce alkaline saliva with pH buffering properties, therefore balancing the pH in the stomach [26,27,28]. Several authors [29,30,31] have studied the slow feeding effect, for example, using hay bags, on the onset of stereotypies and have concluded about the positive effect of these devices. We, therefore, postulate that the differences observed in our study regarding the onset of stereotypies in ration- and ad libitum-fed horses, particularly during the post-feeding period, may have this explanation. Horses feeding quickly on concentrate may have a quick drop in the pH in their stomachs due to an increase in gastric activity not properly compensated by the buffering activity of the saliva. Therefore, a higher frequency of stereotypies was observed in the post-feeding period of ration-fed horses. We, therefore, postulate that the differences observed in our study regarding the onset of stereotypies in ration-fed and ad libitum-fed horses, particularly during the post-feeding period, may be explained by this.

The anticipatory behaviour with an increase in stereotypies being displayed as the feeding time approaches is also much more evident in ration-fed horses, tallying the results obtained by Peters and colleagues [19]. If a horse displays a pessimistic judgment, it is anticipated to demonstrate more behaviours associated with negative states, such as stereotypic behaviours [32] or heightened anticipatory behaviour towards a familiar reward [33]. Therefore, anticipatory behaviour can be used as a measure of animal welfare, as it can be displayed more intensively towards positive reinforcement opportunities [3,34].

Similarly to stereotypies, higher frequencies of arousal behaviours are also observed in the post-feeding period of ration-fed horses. There is an apparent positive correlation between stereotypies and the level of excitement or lack of relaxation observed. Briefer et al. [35] stated that arousal behaviours associated with negative experiences represent an increase in stress. Therefore, the increased arousal in the post-feeding period is associated with higher levels of stress, which also explains the higher frequency of stereotypies. Arousal behaviours also increase in frequency as the feeding time approaches and together with the stereotypies are a good indicator of anticipatory behaviour in ration-fed horses.

The higher frequencies of investigation behaviour in the pre-feeding period were a result also obtained by Peters et al. [19]. The authors explained this result by comparing it with the results obtained while studying other species (e.g., rats [36] and mink [37]), and it is understood as an anticipatory behaviour associated with the expectation of some reward. Concerning the continuation of the behaviour in ration-fed horses in the post-feeding period, it can be explained by the fact that a quick meal opposes the innate behaviour of continuing to forage; therefore, horses maintain the behaviour as if they were still expecting to be rewarded.

Locomotion and maintenance have an apparent negative correlation, as locomotion increases in the feeding period while maintenance decreases. As discussed before, locomotion is associated with feeding, as horses’ wild counterparts travel distances up to 17.9 km/day (up to 7.2 km/day in domestic horses using 16 ha paddocks) [38]. Locomotion, therefore, is also an innate behaviour associated with feeding, and it is not unexpected that we found these results. On the other hand, maintenance behaviours are associated with spare time and relaxation and are displayed more naturally after the satisfaction of the most basic needs, should we use the principle established by Maslow’s Pyramid of human needs applied to horse behaviour as it was applied to dogs by Griffin and colleagues [39].

Previous research has shown that anticipation significantly increases between human cues and feed presentation [19]. The measurement of the timescale of the onset of anticipation without any human cues had not been conducted previously. Within this study, behaviour was monitored for one hour pre feed using CCTV cameras to eliminate human presence altering horses’ behaviour during measurements. The evidence of increased anticipation during a time when no human cues were present suggests that a circadian pattern develops in relation to the regular feeding time, as suggested by Mistlberger [40].

## 5. Conclusions

The behaviours associated with feeding stress are more commonly observed in ration-fed horses and in the post-feeding period. Anticipatory behaviour does increase as regular feeding times approach with the onset measurement revealing a significant increase within the final 20 min of measurement during the approach to regular feed times. Anticipatory behaviour is also exhibited to a greater extent by horses fed a rationed diet in comparison to those fed ad libitum forage, during both the pre- and post-feeding periods. Anticipatory behaviour was significantly lower during baseline measurements.

Anticipation can be stimulated both by the horses’ circadian rhythms or by external cues. Since horses can develop anticipatory behaviours towards scheduled or predictable positive events independently, this behaviour might serve as a less resource-intensive method for animal welfare practitioners to assess the well-being of individual horses on the fly.

## Figures and Tables

**Table 1:** Behaviour categories and elements are considered in this study. The names are self-descriptive. When that is not the case, a description is given within brackets.

Behavioural Category	Behavioural Elements Within the Main Category
Maintenance	Eating, drinking, standing at rest, lying down, urination, defecation, scratching and self-grooming
Locomotion	Walking with a purpose or relaxed
Investigation	Foraging for food, standing alert and investigation of objects within the box
Arousal	Tail swishing, vocal call, head shaking, stamping, striking, threatening face (ears pinned back), buck (buck investigation) and rear (front legs raised, forehand higher than hind quarters)
Stereotypies	Repetitive head shake, repetitive head nod, wind sucking, crib biting, box walking (no purpose or relaxation) or pacing, weaving, pawing and door kicking
Others	Others

Note: Repetitive behaviours were counted once and not one time per repetition. Striking refers to “buck”, and “stamping” was differentiated from “pawing”.

**Table 2:** Poisson regression (log-linear link) models adjusted to the behaviour groups. The adjustment statistics are given by the likelihood Chi-square test, and the degree of adjustment is given by the Akaike’s information criterion (AIC).

Model	Likelihood Ratio Chi-Square Test			AIC
	Chi-Square	df	*p*-Value	
Stereotypical	417	5	<0.001	966
Arousal	109	5	<0.001	210
Investigation	314	5	<0.001	631
Locomotion	75	5	<0.001	271
Maintenance	435	5	<0.001	646

**Table 3:** Poisson regression (log-linear link) models adjusted to the behaviour groups. Parameter estimation. Adjustment statistic given by the Wald Chi-square test.

	Intercept	Feed Type	Feed Period		Interactions	
		AL	Baseline	Post	AL × Base	AL × Post
Stereotypical	2.303 ***	−0.376 **	0.422 **	1.037 ***	−3.447 ***	−2.061 ***
Arousal	NS	NS	NS	1.159 ***	NS	−3.174 ***
Investigation	3.345 ***	0.188 *	−1.246 ***	NS	−1.014 ***	−0.678 ***
Locomotion	1.253 ***	NS	−30.45 ***	−1.496 ***	NS	1.340 *
Maintenance	2.979 ***	−0.395 ***	1.103 ***	0.369 ***	0.359 **	0.621 ***

Notes: in feeding type, “ad libitum-fed” was used as the response and “ration-fed” as the reference; in feeding period, “post” and “baseline” were used as the response and “pre” as the reference; levels of significance, NS *p* > 0.05, * *p* < 0.05, ** *p* < 0.01 and *** *p* < 0.001; the level of significance of the model is represented near the respective behaviour; AL, ad libitum.

## Data Availability

The original data presented in this study are openly available in ResearchGate at https://doi.org/10.13140/RG.2.2.13678.01604.
